# The Clot Thickens: Recent Clues on Hematopoietic Stem Cell Contribution to Age-Related Platelet Biology Open New Questions

**DOI:** 10.20900/agmr20210019

**Published:** 2021-10-28

**Authors:** Donna M. Poscablo, E. Camilla Forsberg

**Affiliations:** 1 Institute for the Biology of Stem Cells, University of California-Santa Cruz, Santa Cruz, CA 95064, USA; 2 Program in Biomedical Sciences and Engineering, Department of Molecular, Cell, and Developmental Biology, University of California-Santa Cruz, Santa Cruz, CA 95064, USA; 3 Biomolecular Engineering, University of California-Santa Cruz, Santa Cruz, CA 95064, USA

**Keywords:** platelets, aging, hematopoietic stem cells, megakaryocyte progenitor cells, hematopoiesis, platelet hyperactivity

## Abstract

Platelets provide life-saving functions by halting external and internal bleeding. There is also a dark side to platelet biology, however. Recent reports provide evidence for increased platelet reactivity during aging of mice and humans, making platelets main suspects in the most prevalent aging-related human pathologies, including cardiovascular diseases, stroke, and cancer. What drives this platelet hyperreactivity during aging? Here, we discuss how hematopoietic stem cell differentiation pathways into the platelet lineage offer avenues to understand the fundamental differences between young and old platelets. Recent advances begin to unravel how the cellular and molecular regulation of the parent hematopoietic stem and progenitor cells likely imbue aging characteristics on the resulting Plt progeny. The resulting mechanistic insights into intrinsic platelet reactivity will provide strategies for selectively targeting age-related pathways. This brief viewpoint focuses on current concepts on aging hematopoiesis and the implications for platelet hyperactivity during aging.

## PLATELET-RELATED DISORDERS DURING AGING

**Figure 1.**
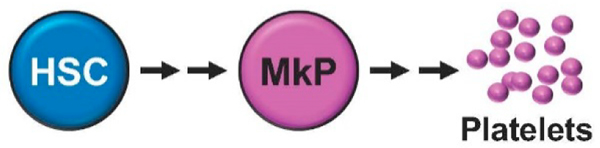
Hematopoietic stem cells (HSCs) give rise to platelets via megakaryocyte progenitors (MkPs). This review focuses on recent reports that have shown that HSC and MkP aging influence the number and function of platelets.

**Figure 2.**
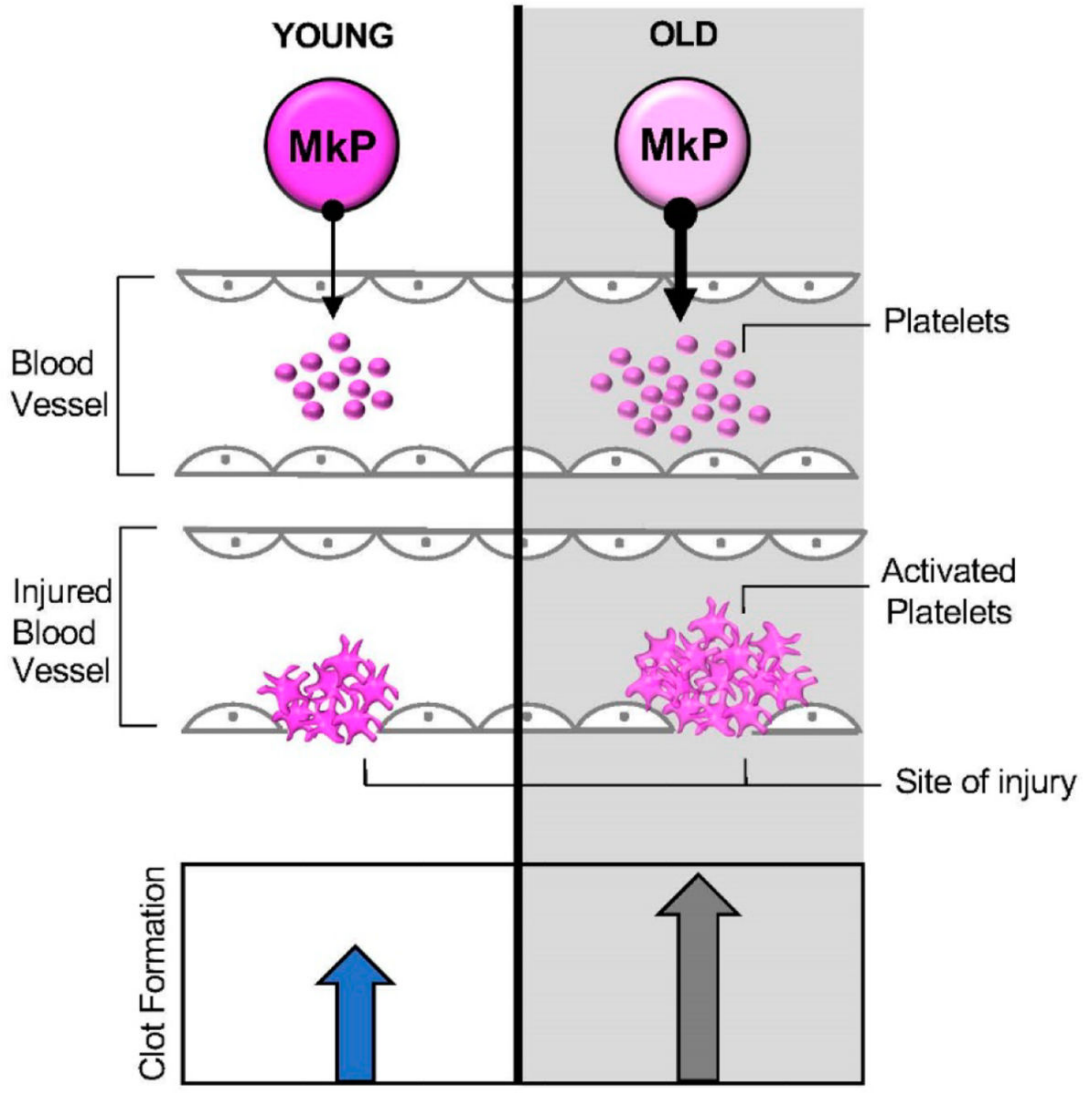
Age-related changes in platelets support prothrombotic state observed in the elderly. Platelets are derived via Megakaryocyte Progenitors (MkP). Upon vascular injury, platelets adhere to the exposed subendothelial matrix, become activated, and aggregate with nearby platelets to form a clot. Mice, and possibly humans, have increased Plt numbers upon aging; both species display Plt hyperreactivity. During aging, MkPs likely give rise to deleterious platelets with functional defects, the culprits of potentially occlusive clots observed in thrombotic disorders that plague the elderly.

Advanced aging is associated with significantly increased incidences of thrombotic diseases and comorbidities. The occurrence of thrombosis in the elderly is linked to age-related differences in platelet (Plt) biology, which may have their origins in Plt precursor cells (Figure 1). Plts are anucleated cell fragments derived from megakaryocytes and are best known for their essential role in hemostasis, the process that stops bleeding while maintaining normal blood flow in the event of vascular injury. Upon damage, circulating Plts adhere to the site of injury, become activated, and promote aggregation to form a Plt plug (Figure 2). These properties make Plts key players in thrombotic disorders. Hemostasis is frequently compromised with age, and both thrombocytosis (too many Plts) and thrombocytopenia (too few Plts) lead to increased mortality in the elderly [1–6]. Consequently, millions of people take prophylactic anti-Plt therapies for long periods of time to reduce Plt count (by reducing agents such as hydroxyurea, anagrelide) or to inhibit Plt function (by antithrombotic agents such as aspirin) in order to reduce the risk of morbidity and mortality associated with occlusive thrombosis [7–9]. Despite the success of anti-Plt therapies, thrombotic diseases remain a leading cause of death, in part due to complications of anti-Plt therapies [2]. Due to the essential role of Plts in hemostasis, patients treated with anti-Plt drugs also face a risk of increased bleeding; therefore, alternative or more refined anti-Plt therapies are needed to balance the thrombotic risk and the subsequent risk of serious bleeding [7]. Targeted therapeutics are also needed in the disease states that require an increase in Plt count or accelerated Plt activity. Current treatments may include prophylactic Plt transfusion, antifibrinolytic agents (such as aminocaproic acid or tranexamic acid), or factor replacement therapy. Together, the numerous Plt-related disorders along with the increasing life expectancy of human populations underscores the profound financial and clinical burden imposed by age-related Plt diseases. Thus, the advancement of therapies that control Plt production and function by an improved understanding of the mechanisms of age-related Plt biology is a critical patient and public health goal.

## NUMERICAL, MOLECULAR, AND FUNCTIONAL CHANGES TO PLATELETS DURING AGING

Aging is accompanied by changes to Plt biology. Epidemiological studies of Plt numbers remain inconclusive: some studies observed no differences between the young and elderly populations, while others suggest that Plt numbers are decreased in the elderly population compared to their younger counterparts [10–12]. This inconsistency is perhaps in part due to intra-individual variation during human aging [13,14]. In murine studies, conversely, there is a consistently observed increase in Plt count in old mice [6,15–17]. Despite these discrepancies between the abundance of Plts in aged humans and mice, both mouse and human aging are associated with Plt hyperreactivity. Therefore, translation of murine studies to human application will likely emerge from an understanding of the consequences of aging on Plt function. There are clear age-related changes to both Plt generation and function (Figure 2). Several in vitro studies of human Plts have reported an increase in aggregability, which is inversely associated with decreased bleeding time in the elderly [1,18,19]. These observations suggest faster clot formation upon aging, and that this enhanced Plt activity is a biomarker of thrombosis in humans. There is also evidence for potential molecular regulators of age-associated changes to Plt function. For example, sequencing analysis revealed differences in mRNA and microRNA expression patterns between young and old human Plts [20]. As in other tissues, oxidative stress in Plts has also been shown to increase during aging [6,21]. Another important line of evidence demonstrated that the physiological agonists presented by an inflammatory state enhances age-related Plt reactivity and thrombosis [15]. These prothrombotic mechanisms appear intrinsically propagated by aged Plts even in the absence of the agonists [17]. These findings highlight the need to understand how distinct cellular and molecular hallmarks of aging drive changes to Plt function.

## HEMATOPOIETIC STEM CELL CONTRIBUTION TO PLATELET AGING

Aging of the hematopoietic system has been the focus of intense investigation for decades, yet there remains a significant need to link mechanisms of hematopoiesis to the biology of their Plt progeny. Advanced age is associated with dysregulation in both the number and function of blood and immune cells. Due to the short half-life of circulating hematopoietic cells, including Plts, they are continuously produced from Hematopoietic Stem Cells (HSCs) via progenitor cells that primarily reside within the bone marrow (BM) (Figure 1). The short lifespan of mature cells means that they likely do not undergo true aging themselves, but “inherit” age-related properties from their parent stem and progenitor cells. Given that HSCs are at the top of the hematopoietic hierarchy, much focus has been directed towards understanding how aging affects HSCs [15,22–24]. Although HSC numbers increase with age, they exhibit functional decline when tested in transplantation experiments: old HSCs show less robust hematopoietic reconstitution efficiency in recipient mice relative to young HSCs. Despite comprising ~99% of all mature hematopoietic cells, Plts and red blood cells (RBCs) have been ignored in the vast majority of HSC transplantation studies due to technical limitations in distinguishing donor- from host-derived Plts and RBCs [25]. The Plt and RBC potential of HSCs and progenitor cells has therefore more often been investigated by in vitro differentiation assays rather than by in vivo studies. Development of fluorescent transgenic mice such as Ubc-GFP and KuO mice that harbor fluorescent Plts and RBCs has rectified the technical limitations with transplantation experiments because these models allow direct tracking of donor-derived Plts and RBCs [25–29]. Additionally, recent barcoding studies include endpoint analyses of the immediate progenitors of Plts and RBCs [30]. Implementation of these tools has led to exciting findings, including the reported existence of self-renewing Plt-restricted or Plt-biased cells [26,31–33]. However, the differentiation paths, abundance, regulation, and functional significance of these putative, lineage-restricted but self-renewing, cells are currently unclear. At present, our understanding of HSC differentiation into Plts and RBCs lag significantly behind other lineages, particularly in the context of aging.

## ENHANCED FUNCTION OF OLD MEGAKARYOCYTE PROGENITOR CELLS: IMPLICATIONS FOR AGE-RELATED PLATELET HYPERREACTIVITY

**Figure 3.**
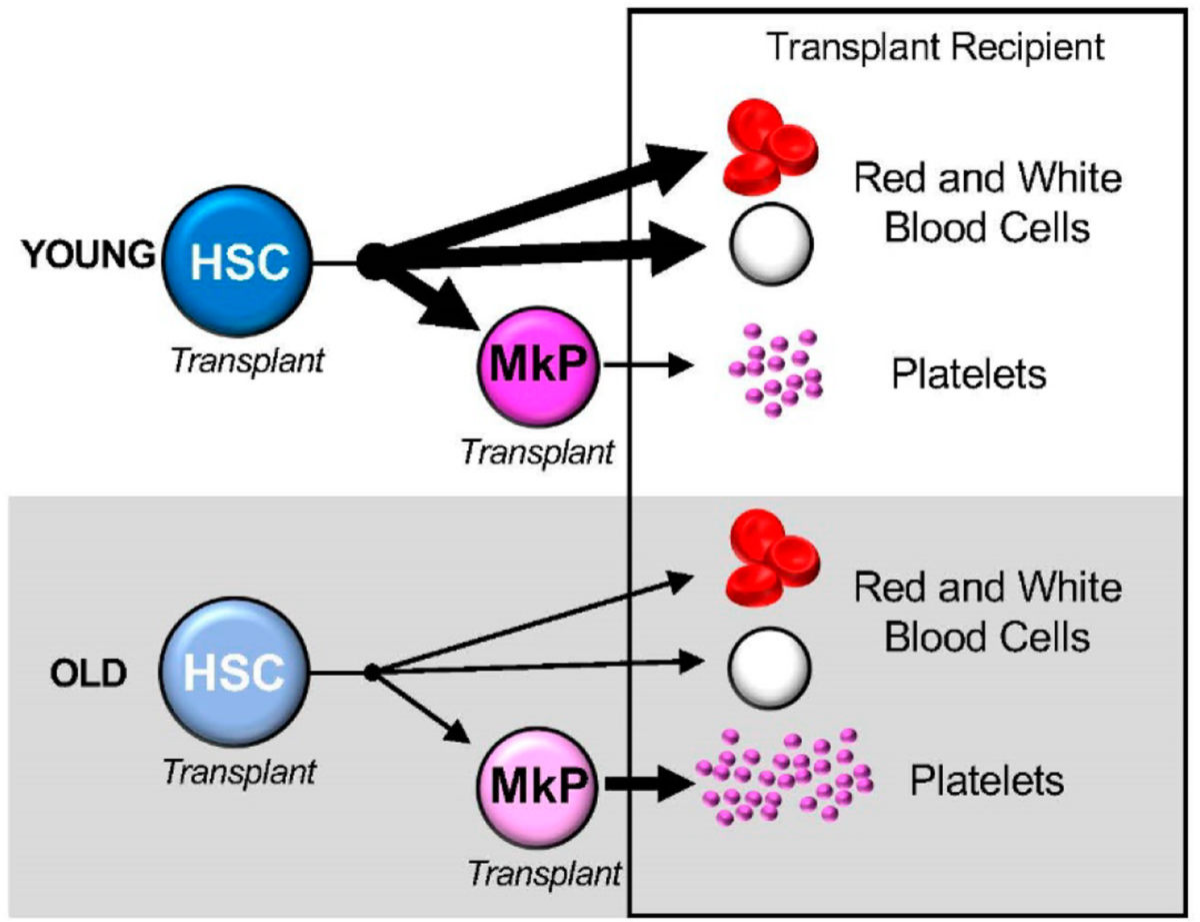
Hematopoietic stem and progenitor cell origins of platelets are altered during aging. Transplantation experiments revealed that old Hematopoietic Stem Cells (HSCs) exhibited a decline in generation of all hematopoietic lineages, including Megakaryocyte Progenitors (MkPs) and platelets. However, old MkPs exhibited a remarkably greater expansion capacity upon aging, giving rise to significantly greater numbers of platelets compared to young MkPs.

Our recent discoveries on the mechanisms of aging megakaryopoiesis advanced our understanding of the hematopoietic BM origins of Plt biology during aging [15]. In this study, we investigated the regulation of Plt production modulated by HSCs and megakaryocyte-committed progenitor cells (MkPs). In old mice, the expansion of HSCs was accompanied by an expansion of MkPs and their descendant Plts. To determine how intrinsic and extrinsic factors influence Plt production from aged HSCs, we transplanted young HSCs into young and old recipient mice, and vice versa. Interestingly, these experiments revealed that old HSCs did not generate a selective increase in the number of MkPs and Plts, as in unmanipulated aged mice (Figures 2 and 3) [15,17,34]. These observations raised our curiosity around aging MkPs. Given the reconstitution deficit displayed by old HSCs, we reasoned that MkPs would also display functional deficiency. Surprisingly, old MkPs displayed a remarkable capacity to engraft into recipient mice by generating greater Plt numbers compared to young MkPs (Figure 3). Our in vitro analysis also demonstrated greater expansion capacity of old MkPs. Importantly, RNA sequencing of young and old MkPs revealed age-related changes in gene expression profiles, including changes in genes involved in Plt production and function and in bleeding disorders. Interestingly, a few of the genes upregulated in old MkPs are also implicated in acute myeloid leukemia, including *Pbx3*, *Lair1*, and *Mllt3* [35–37]. These changes in the transcriptome of old MkPs support a model in which MkPs propagate age-related dysregulation of Plt biology. Therefore, our study revealed novel cellular and molecular mechanisms of age-related alterations to megakaryopoiesis. In addition to the chronic stress presented by aging, acute stress condition has been shown to influence MkPs. Upon infection, acute inflammation drives MkP maturation and increases Plt counts in young mice [32]. Together, these findings may help to explain intrinsic and extrinsic regulation of MkPs during youthful and aging megakaryopoiesis. Furthermore, given that both the increase in Plt numbers and hyperactivity appear to play a significant role in the increase of thrombotic risk during aging, an important next step is to determine how age-related alterations to MkPs may poise their descendent Plts for activation and aggregation.

Collectively, the new insights from our group and others on aging megakaryopoiesis shed light on the mechanisms of aging by which Plts “inherit” their properties from their parent stem and progenitor cells (Figure 1). While HSC Plt potential declines during aging, MkPs gain a remarkable capacity to contribute to Plt production. These functional alterations may dictate the etiology of Plt-related disorders during aging and provide therapeutic avenues for manipulating hematopoietic stem and progenitor cells to control hemostasis throughout life. Unraveling the specific contributions of hematopoietic stem and progenitor cells to consequential Plt production and function will be critical to the success of ameliorating and treating life-threatening Plt disorders that accompany aging.
